# Novel Strategies in the Prevention and Treatment of Urinary Tract Infections

**DOI:** 10.3390/pathogens5010013

**Published:** 2016-01-27

**Authors:** Petra Lüthje, Annelie Brauner

**Affiliations:** Department of Microbiology, Tumor and Cell Biology, Division of Clinical Microbiology, Karolinska Institutet and Karolinska University Hospital, Stockholm SE-171 76, Sweden; Petra.Luthje@ki.se

**Keywords:** urinary tract infection, antimicrobial peptides, estrogen, vitamin D, herbal extracts

## Abstract

Urinary tract infections are one of the most common bacterial infections, especially in women and children, frequently treated with antibiotics. The alarming increase in antibiotic resistance is a global threat to future treatment of infections. Therefore, alternative strategies are urgently needed. The innate immune system plays a fundamental role in protecting the urinary tract from infections. Antimicrobial peptides form an important part of the innate immunity. They are produced by epithelial cells and neutrophils and defend the urinary tract against invading bacteria. Since efficient resistance mechanisms have not evolved among bacterial pathogens, much effort has been put into exploring the role of antimicrobial peptides and possibilities to utilize them in clinical practice. Here, we describe the impact of antimicrobial peptides in the urinary tract and ways to enhance the production by hormones like vitamin D and estrogen. We also discuss the potential of medicinal herbs to be used in the prophylaxis and the treatment of urinary tract infections.

## 1. Introduction

Urinary tract infections belong to the most common infections worldwide; more than 50% of women will experience at least one UTI during her lifetime [1]. Even though often self-limiting and seldom developing into more severe infections, cystitis causes considerable costs for the patient and the public health system. Moreover, in a substantial number of women, infections will recur several times during the year. While in adults, in the absence of pre-disposing factors, women are several times more often affected than men [2], the gender distribution among children varies with age, with boys being most susceptible as infants below 12 months of age [3,4,5]. The cumulative incidence during the first six years of life however is almost 7% in girls and close to 2% in boys [6]. Most infections have good prognosis, but the risk for renal scarring might be as high as 40%, largely dependent on the diagnostic method used [7,8,9,10]. Similar to UTI in women, there is a risk for recurrence in 25%–40% of children with UTI [11,12].

Acute infections are commonly treated with antibiotics, and antimicrobial prophylaxis might be considered for women suffering from recurrent infections. The most common uropathogen is *Escherichia coli* (*E. coli*), accounting for >80% of uncomplicated UTIs. While highly resistant strains are yet not common in countries with relatively strict antibiotic regimes, multi-drug resistance and extended-beta-lactamase (ESBL) resistance is frequently detected in uropathogenic *E. coli* (UPEC) isolated in countries with less controlled antibiotic usage [13,14].

Even though bacteria have evolved mechanisms to resist the antimicrobial effect of endogenous antibiotics [15,16], resistance to these antimicrobial peptides (AMPs) has not effectively developed. The classical AMPs have common features, as they are small polypeptides of a few amino acids, are cationic and amphipathic. The highly positive charge explains the relatively selective action on bacterial membranes, which are more negatively charged compared to mammalian cell membranes [17]. The interaction with the peptides leads to membrane disruption and eventually lysis of the cell. However, in addition to the direct antibacterial activity, AMPs exhibit alternative functions supporting the immune system [18]. This might be immuno-modulatory properties [19] or actions on cell differentiation and tissue remodeling, which ultimately strengthen mechanical defense of epithelial barriers [20,21,22]. This wide range of actions together with the apparently low risk for resistance development make AMPs interesting mediators for novel treatment strategies. In this review, AMPs in the urinary tract will be presented, and different strategies to enhance innate immunity by AMPs or other epithelial-located mechanisms will be discussed.

## 2. Recurrent UTI

While the acute infection is self-limiting within a few days and is still easily treated with antibiotics, in a considerable number of women, the infection may recur within weeks or months. Recurrent UTI is defined by two or more infections within six months or at least three infections during a year. The recurring infection is often caused by the same bacterial strain as a previous infection, indicating that the bacterium is persisting in the body despite treatment with a suitable antibiotic [23]. Both the perianal and the vaginal flora serve as reservoirs for infections of the urinary tract [24,25,26]; however, *E. coli* appears to persist even in the bladder tissue. When entering the bladder, *E. coli* adheres to the bladder epithelium, and a small proportion of bacteria invades the cells. In this niche, protected from mechanical and cellular immune defense, bacteria multiply rapidly to form so-called intracellular bacterial communities (IBCs) [27,28]. As part of the defense mechanism, infected cells undergo apoptosis, lose contact with the neighboring cells and are shed and excreted with the urine [29,30]. Bacteria released from IBCs can therefore invade cells of deeper layers, which became exposed upon exfoliation of the outer layer of umbrella cells [28]. Within these cells, bacteria do not multiply, but instead form small dormant colonies, referred to as quiescent intracellular reservoirs (QIRs). While the signals inducing re-activation of these bacteria are not fully elucidated yet [31], QIRs are considered the source of recurrent infections [32,33]. Even though this model is based on studies in mice, there is an indication that a similar infection cycle takes place in women [34,35]. IBCs have also been detected in the urine of children with recurrent UTI [36,37]. However, especially in this patient group, other pathomechanisms, in particular vesicoureteral reflux [38,39] and genetic predisposition (reviewed in [40]), have to be considered.

Intravesical bacterial persistence explains the relative ineffectiveness of antimicrobials to prevent recurrence of the infection, since intracellular and metabolically-inactive bacteria make a difficult target for conventional antibiotics and the body’s own defense. Moreover, these intracellular bacteria reside in biofilm-like organizations and express many factors commonly associated with biofilm formation on abiotic surfaces *in vitro*. The non-fimbrial adhesin antigen 43 (Ag43) has been detected in IBCs in mice [27], and a particular variant of this protein, Ag43a_CFT073_, was shown to promote persistence in the mouse urinary tract [41]. In a collection of *E. coli* from children with recurrent UTIs, the presence of *agn43* was linked to persisting strains [42]. Infections with strains carrying the gene coding for Ag43a_CFT073_ in particular caused a lower immune response in these children (as determined by urinary IL-8 levels) compared to strains lacking this gene. This finding might indicate that immune evasion, either during direct contact with the host cell or by more efficient formation of biofilm intracellularly, promotes the persistence of these strains. The major *E. coli* biofilm components curli and cellulose contribute similarly to the persistence of UPEC in the urinary bladder. In women suffering from recurrent UTIs, persisting strains were more likely to express both factors than strains causing sporadic infections [25]. In a mouse UTI model, reduced immune induction by strains producing cellulose promoted their persistence in the tissue, while curli fimbriae supported early virulence of the strain by mediating adherence [43].

While treatment with conventional antimicrobial agents is effective against acute UTI, it does not prevent the recurrence of the infection, because intracellular, dormant bacteria are not eradicated. Long-term prophylaxis with low doses of antibiotics might efficiently inhibit the emergence of intravesical bacteria during the treatment period [44,45], but infections are likely to re-occur when the prophylactic treatment is terminated [46,47]. Therefore, alternative prophylactic and therapeutic strategies are needed, especially for women suffering from recurrent UTI [48].

## 3. Antimicrobial Peptides in the Protection of the Urinary Tract

Antimicrobial peptides are produced by immune cells, but also expressed by epithelial cells throughout the body. They confer instant protection by their antimicrobial activity against a wide range of pathogens, but contribute also by immune-modulatory properties or alternative actions on the pathogens. Several AMPs have been described in the lower and upper urinary tract [49,50]. We have previously demonstrated the importance of the human cathelicidin LL-37/hCAP18 for the protection of the urinary tract [51], and recent research has established a major contribution of ribonuclease (RNase) 7 to the urinary defense against uropathogens [52,53]. The expression of human β-defensins (hBDs) in the urogenital tract is well established [54,55], but their role during UTI is only incompletely understood. hBD1 is constitutively expressed in various epithelia throughout the body [56], with high expression levels in the kidney [57] and lower expression in epithelial cells of the urinary bladder [58]. Despite anti-*E. coli* activity of the AMP in urine [57], hBD1 deficiency in mice was not accompanied with increased bacterial burden in bladder or kidneys [58]. Studies with human patients suggest that hBD1 might play a role during acute pyelonephritis [59], while its role in the defense of the lower urinary tract is less certain [60]. In contrast to the constitutive expression pattern of hBD1, hBD2 is expressed only in response to inflammatory stimuli [61] and has been detected in chronically-infected kidneys [62].

Another group of defensins, the α-defensins, have mainly been investigated in the intestinal tract. Recently, however, the presence and activity of the human α-defensin 5 has been demonstrated also in the urinary tract [63]. Other peptides inhibit bacteria by nutrient depletion, most importantly iron. Hepcidin is a major regulator of iron homeostasis, but may also exhibit direct antibacterial activity against *E. coli* [64]. Lipocalin 2 and lactoferrin influence bacterial growth by depletion of essential metals, such as iron. Both proteins are stored in neutrophil granules, but are also expressed by epithelia of the urinary tract, primarily in the kidney [65]. Renal lipocalin 2 is upregulated upon infection, secreted with the urine and, thus, contributes to the defense also of the lower urinary tract [66,67]. A similar antibacterial mechanism has been suggested for the S100 protein psoriasin via depletion of zinc [68]. Despite its pronounced activity against *E. coli* and documented expression in urinary tissues [69,70], the contribution of psoriasin to the urinary defense has not been investigated. Finally, the Tamm-Horsfall protein, or uromodulin, a renal-specific protein, which is secreted with the urine at high concentrations, fends off *E. coli* and other pathogens by interfering with the adhesion to the host cells of the lower urinary tract [71]. This review will mainly focus on the human cathelicidin LL-37/hCAP18, its role in urinary protection and its interaction with *E. coli*.

### 3.1. The Role of Cathelicidin in the Urinary Tract

Similar to other AMPs, the human cathelicidin is produced as an inactive precursor, containing a signal peptide and the propeptide hCAP18 [72,73]. After cleavage, the antimicrobially-active peptide consists of 37 amino acids starting with two lysin residues, therefore referred to as LL-37. Further cleavage products with distinct antimicrobial activities might be generated at different sites of the body [74,75,76]. In mice, the homologues peptide is referred to as CRAMP, cathelicidin-related AMP [77]. Since much of our knowledge is based on experiments in mice and analyses of mouse tissues, the precise role of LL-37/hCAP18 in the human urinary tract can only be estimated.

The sensitivity of *E. coli* strains isolated from the urinary tract differs. Interestingly, strains isolated from more severe infections, *i.e.*, pyelonephritis, exhibit decreased sensitivity to LL-37 *in vitro* compared to less invasive strains isolated from cases of cystitis [51]. Several factors expressed by UPEC interfere with the bactericidal action of LL-37. Curli fimbriae (adhesins and biofilm component of *E. coli*) bind to LL-37 and thus prevent interaction with the bacterial cell membrane ([43], discussed below). Biofilm formation *per se* might moreover hamper LL-37 penetration and thus impair its action also in a widely unspecific manner. The outer membrane protease OmpT has been shown to contribute to resistance against LL-37 in enteric *E. coli* pathotypes by degrading the peptide [78]. The UPEC strain CFT073 also expresses OmpT with proteolytic activity against LL-37; the contribution to protection against LL-37 is however less clear [79]. In various Gram-negative bacteria, modification of the outer membrane has been implicated in protection against LL-37 [16]. Changes in the lipid A moiety of the lipopolysaccharide (LPS) can partly neutralize the negative charges and thus renders the surface less susceptible for binding cationic AMPs [80]. As part of the immune-modulatory function of LL-37 is based on its LPS-neutralizing effect [81], a correlating function of LPS in the protection of *E. coli* against this AMP may be anticipated. However, in *E. coli*, this has not been studied in detail.

LL-37/hCAP18 is expressed throughout the urinary tract. Epithelial cells of the lower and upper urinary tract express the peptide constitutively at low levels. Higher levels are produced in response to bacteria and by neutrophils recruited during infection, as reflected by increased urinary levels in patients with UTI compared to healthy controls [51,60]. In a mouse model of ascending pyelonephritis, morbidity and mortality were higher in animals deficient for CRAMP compared to wild-type, with higher bacterial titers both in the kidneys and the bladder [51]. Experiments with neutrophil-depleted mice demonstrated that epithelial-derived LL-37 influenced morbidity and bacterial load in the bladder, while neutrophil-derived LL-37 reduced renal bacteria and mortality and inhibited systemic spread of the infection. Neutrophilic LL-37 contributes to intracellular killing of bacteria after phagocytosis, but is also part of neutrophil extracellular traps (NETs), a net of fibers consisting of chromatin and granular proteins released from neutrophils [82]. Rather than killing entrapped pathogens directly, LL-37 appears to promote NET formation and increases NET stability [83,84]. The importance of NETs and the role of LL-37 in this context have not been investigated in the urinary tract so far.

In a mouse strain resistant to vesicoureteral reflux, CRAMP deficiency did not result in higher bacterial titers [85]. Instead, the pro-inflammatory activity of CRAMP resulted in more severe tissue damage and higher bacterial loads in wild-type mice during acute infection. Similar observations were made when LL-37 was directly instilled in the urinary bladder of mice [86,87]. As indicated above, curli fimbriae trap LL-37, preventing it from exerting its bactericidal activity at the bacterial outer membrane [43]. While the non-curliated pyelonephritis strain *E. coli* CFT073 was used in the first study by Chromek *et al.* [51], the curliated cystitis strain UTI89 was used in the study by Danka *et al.* [85]. Due to its curli fimbriae, this strain may be less sensitive to LL-37 (or CRAMP, respectively). Bacterial survival in the bladder might thus not be that much affected by this AMP; instead, the pro-inflammatory activity of CRAMP may be dominating the histopathological picture. In co-infection experiments with isogenic *E. coli* strains, a curli-expressing wild-type strain was recovered in higher numbers compared to a biofilm-deficient strain lacking curli fimbriae [88]. While this might primarily be due to a deficiency in the formation of IBCs, increased sensitivity of the non-curliated strain towards LL-37 may contribute.

Neutrophil-derived CRAMP was of major importance in the protection of the upper urinary tract and systemic spread of the infection [51]. The role of CRAMP in the acute protection of the urinary bladder is less evident and might to larger extent depend on the infecting strain [51,85]. It can also not be ruled out that CRAMP has an impact on long-term intracellular bacterial persistence and thus recurrence of infections. Lower baseline levels of urinary LL-37 in women who had had a UTI compared to baseline levels from women who never had one indicate that CAMP plays a role in the protection of the lower urinary tract [60].

### 3.2. Interaction of LL-37 with Bacterial Biofilm

At sub-inhibitory concentrations, LL-37 has been shown to influence biofilm formation of different bacterial species [43,89,90,91,92,93,94], including species with potential relevance in the urinary tract. The direct interaction of LL-37 with microbial surface structures may be responsible in some cases [43,94], including the opportunistic fungal pathogen *Candida albicans* [95]. In *Pseudomonas aeruginosa*, LL-37 reduced biofilm formation by impacting quorum sensing systems, leading to decreased bacterial attachment and increased twitching motility [89].

In *E. coli*, curli-dependent biofilm is inhibited by LL-37 through direct interaction of the AMP with CsgA, the major subunit of curli fibers [43]. By binding of LL-37 to monomeric CsgA, it inhibits the polymerization and thereby the curli formation and ultimately reduces biofilm formation. However, binding of LL-37 to already established curli fimbriae at the bacterial surface neutralizes the bactericidal effect of the peptide.

## 4. Supporting Endogenous Defense Mechanisms

Since prophylactic antibiotic treatment is unsatisfying and moreover bears the risk for selecting for resistant strains, alternative approaches have been investigated. One attractive strategy is to enhance endogenous defense mechanisms, which will be well tolerated by the host organism while at the same time are less likely to induce resistance in the pathogen.

### 4.1. Vitamin D

The implication of vitamin D on the immune system is well established and has empirically been employed by the usage of sunlight to fight infections, such as tuberculosis [96]. More recently, a direct linkage of vitamin D and CAMP expression was identified as one underlying mechanism [97]. The promoter region of the *CAMP* gene contains a vitamin D-responsive element (VDRE), which binds the receptor in a ligand-dependent and -independent manner, but transcription efficacy is greatly enhanced in the presence of vitamin D. This genetic organization is restricted to primates, including humans, and not present in rodents, which hampers animal studies in this field.

The action of vitamin D requires the hormonally-active form 1,25-dihydroxyvitamin D_3_. Vitamin D precursors obtained by exposure to sunlight or with the diet are converted to the major storage form 25-hydroxyvitamin D_3_ in the liver [98], while the kidney is the principal organ to convert the inactive form into active 1,25-dihydroxyvitamin D_3_ by the 1α-hydroxylase (Cyp27B1) [99]. However, a number of other tissues, including epithelial cells, at different body sites express this enzyme and are able to locally activate vitamin D. The Cyp27B1 enzyme could also be detected in bladder epithelial cells [100]. Upregulation of the vitamin D inactivating enzyme 24-hydroxylase (Cyp24A1) upon exposure to 25-hydroxyvitamin D_3_ indicated furthermore activation of a negative feedback response, indicating the local generation of 1,25-dihydroxyvitamin D_3_.

Several clinical studies have demonstrated the importance of sufficient vitamin D levels for the protection of the urinary tract against infection. In children with UTI, serum vitamin D was significantly lower compared to healthy controls who never had a UTI [101]; moreover, vitamin D levels correlated with the severity of the infection. Similarly, in premenopausal women, low levels of serum vitamin D were associated with a higher risk for recurrent UTI [102]. In an experimental study, we could show that oral vitamin D supplementation was able to increase CAMP production in the human bladder epithelium in response to *E. coli* [100]. Together, these studies clearly demonstrate that vitamin D supplementation provides a potent alternative in the protection against UTI in vulnerable patient groups.

### 4.2. Estrogen

Clinical observations indicate a complex impact of estrogen on UTI pathogenesis. In young women, high estrogen levels are linked to increased receptivity for *E. coli* infection [103]; in women post-menopause who suffer from recurrent UTI, estrogen supplementation might, on the other hand, improve the condition [104,105,106]. Recently, we were able to provide explanations for the seemingly conflicting findings with respect to the estrogenic action in the urinary tract, based on experimental data from animal and *in vitro* models and patients’ material [69]. In the presence of high estrogen levels, corresponding to those found in menstruating women, expression of uroplakin (UP) Ia and β1 integrin, major host receptors mediating *E. coli* adherence and invasion of the uroepithelium [107,108], were elevated, partly explaining the high incidence of UTIs when estrogen levels are high. On the other hand, estrogen caused increased expression of at least three of five tested AMPs in 75% of postmenopausal women after local supplementation with estrogen for two weeks, with the most prominent overall effect on hBD3 [69]. Expression of proteins involved in cell-cell contact was likewise elevated, indicating improved epithelial integrity in the presence of estrogen. Similar to vitamin D, estrogen acts via its receptors and estrogen responsive elements in the gene promoter. The universal effect of estrogen on AMP expression, however, suggests rather an indirect effect linked to estrogen-promoted differentiation of the uroepithelium. In the absence of estrogen, defective differentiation of bladder epithelial cells was noted [109], supporting this hypothesis. The epithelial-protective effect by estrogen might furthermore be supported by dampening the pro-inflammatory response [109], which always coincides with tissue damage. Increased epithelial integrity and higher expression of AMPs as a consequence of estrogenic action on the uroepithelium may together reduce the formation of QIRs as the source of recurrent infections [69,109].

Estrogen levels are high also during pregnancy. While there are no solid data indicating differences in the general receptivity to UTI in pregnant women, an increased risk for ascending infections is well established [110]. Anatomical alterations, such as ureteral dilatation and decreased tone in the bladder and ureter, facilitate ureterovesical reflux and ascendance of bacteria from the bladder to the kidneys. These complex anatomical, physiological and hormonal changes might dominate over-protective estrogenic effects on epithelial defense strategies during pregnancy.

### 4.3. Medicinal Herbs

Herbal preparations are often part of alternative treatment strategies. The active substance or the way of action has not always been identified; thus, treatment is often empirically and without proven effect. However, efforts are made to understand the mechanisms of these medicines, to evaluate their value as alternatives for conventional antibiotic treatment [111].

Some herbal preparations exhibit direct antibacterial activity against uropathogens, at least *in vitro* [112,113,114]. A commercial preparation consisting of *Armoraciae rusticanae* (horseradish) and *Tropaeoli majoris* (nasturtium) was tested in a clinical trial and found effective for prophylaxis against recurrent UTI [115]. *In vitro*, the preparation exhibited antibacterial activity against a wide range of bacterial pathogens, including *E. coli* [112]. A systematic screening of plant extracts traditionally used for UTI treatment showed that several substances did not exhibit direct antimicrobial actions, but interfered with bacterial adhesion to the host cell [116]. Two herbal extracts, *Agropyron repens* (couch grass) and *Zea mays* (maize, corn), acted on the bacteria directly. Direct interference with adhesion of P-fimbriated bacteria has also been proposed for the protective mechanism of cranberries [117,118]. Cranberry products have widely been used for the treatment and prevention of (recurrent) UTIs, but the benefit is discussed controversially [119,120].

Three further plant extracts investigated in the study by Rafsanjany *et al.* [116], *i.e.*, extracts from *Betula* spp. (birch), *Orthosiphon stamineus* (Java tea) and *Urtica* spp. (nettles), acted on the host cell rather than the bacterium to diminish adherence of the pathogen. Such infection-reducing effects have been observed and investigated for other medicinal plants with traditional anti-infective applications. Decoction of *Lactuca indica* (Indian lettuce), a Vietnamese herbal plant with anti-inflammatory and diuretic activity, reduced bacterial infection by interfering with host cell signaling pathways leading to bacterial uptake [121]. Extracts from the Malaysian flowering plant *Labisia pumila* var. *alata* [122] and the seeds from *Citrus reticulata* Blanco (mandarin) [123] reduced bacterial invasion of bladder epithelial cells by downregulation of the invasion-mediating receptor β1 integrin. In addition to the anti-adhesive effect, *Labisia pumila* var. *alata* extracts supported apoptosis-dependent exfoliation of infected cells, but prevented progressive cell death during infection. These actions in combination may reduce the bacterial burden in the upper epithelial cell layer of the urinary bladder while protecting the underlying tissue, prone to developing persisting bacterial reservoirs.

Many medicinal herbs exhibit also anti-inflammatory, analgetic and diuretic effects, which may contribute to their beneficial effect during UTI. A double-blinded, randomized clinical trial comparing symptomatic (ibuprofen) and antibiotic treatments (ciprofloxacin) of women with acute UTI suggested that symptomatic treatment might often be sufficient for the management of most uncomplicated UTI [124]. Thus, anti-inflammatory and analgetic actions might explain the relief by the intake of herbal preparations where direct modes of action are yet unidentified. For example, *Gynostemma pentaphyllum*, mostly known for its anti-diabetic effect, reduced the bacteria-induced inflammatory response in the urinary bladder of rats receiving the herbal extract [125]. Interestingly, *Gynostemma pentaphyllum* also modulated expression of several AMPs. In particular, bladder epithelial cells from treated rats responded with a stronger upregulation of psoriasin, an AMP with strong activity against *E. coli*, compared to control animals.

Even though the action of most traditional herbal medicines is incompletely understood today and placebo-controlled clinical trials are sparse or missing, these studies demonstrate the complex and multifunctional mode of action. Identifying the active substances and establishing their activity will help to develop novel treatment and prophylactic strategies against UTIs.

## 5. Concluding Remarks

Uncomplicated UTIs are often self-limiting, still a frequent indication for antimicrobial therapy. This conventional treatment is usually effective against the acute infection, but long-term usage of antibiotics is required to reduce recurrences. The imperfect protection against recurrent UTIs and the risk of provoking resistance among the bacterial pathogen are two reasons asking for alternatives in the management of UTIs. A promising possibility is the exploitation of the innate immune defense by supporting endogenous mechanisms. The targeted application of natural substances identified in traditionally-used herbal medicines and the knowledge about their way of action provide another source for the development of alternative strategies.
